# Human foot form and function: variable and versatile, yet sufficiently related to predict function from form

**DOI:** 10.1098/rspb.2023.2543

**Published:** 2024-01-10

**Authors:** Robert W. Schuster, Andrew G. Cresswell, Luke A. Kelly

**Affiliations:** ^1^ School of Human Movement and Nutrition Sciences, The University of Queensland, Saint Lucia, Queensland, 4067, Australia; ^2^ Griffith Centre of Biomedical and Rehabilitation Engineering, Griffith University, Gold Coast, Queensland, 4215, Australia; ^3^ School of Health Sciences and Social Work, Griffith University, Gold Coast, Queensland, 4215, Australia

**Keywords:** foot morphology, foot mechanics, longitudinal arch, transverse arch, shape–function modelling

## Abstract

The human foot is a complex structure that plays an important role in our capacity for upright locomotion. Comparisons of our feet with those of our closest extinct and extant relatives have linked shape features (e.g. the longitudinal and transverse arches, heel size and toe length) to specific mechanical functions. However, foot shape varies widely across the human population, so it remains unclear if and how specific shape variants are related to locomotor mechanics. Here we constructed a statistical shape–function model (SFM) from 100 healthy participants to directly explore the relationship between the shape and function of our feet. We also examined if we could predict the joint motion and moments occurring within a person's foot during locomotion based purely on shape features. The SFM revealed that the longitudinal and transverse arches, relative foot proportions and toe shape along with their associated joint mechanics were most variable. However, each of these only accounted for small proportions of the overall variation in shape, deformation and joint mechanics, most likely owing to the high structural complexity of the foot. Nevertheless, a leave-one-out analysis showed that the SFM can accurately predict joint mechanics of a novel foot, based on its shape and deformation.

## 1. Introduction

The observation that the form and function of some biological systems seem to be closely linked has long prevailed in the field of evolutionary biology. This idea was fundamental to the first coherent theory of evolution by Lamarck [1] and the seminal theory of natural selection by Darwin & Wallace [2], and remains an important component of the more recent modern synthesis [3] and its successors. The wide range of examples found in nature that suggest the existence of straightforward form–function relationships entice the assumption that this must be true for most biological structures. Following this logic, investigators have tried to identify specific anatomical adaptations for the unique effectiveness of our bipedal gait. A common method used in this endeavour is comparing our anatomy with that of our closest extinct and extant relatives, with the aim of isolating the distinctive features that set us apart. Such comparisons have produced a comprehensive list, ranging from our relatively flat face, spinal curvature, short and narrow pelvis, large hip and knee joints, long legs and short arms to the unique shape and structure of our feet [4,5].

Specifically, our feet have received considerable attention. Despite their significance as our most common interface with the ground, their highly complex structure has made deciphering the relationship between foot form and function exceedingly challenging. Consequently, various form–function relationships concerning modern human feet have been proposed. Early discussions on the functional advantages our feet provide identified their arched configuration; particularly the medial longitudinal arch (MLA). This MLA was recognized as characteristic of modern humans and thought to reflect the forces transmitted and leverage provided by the foot [6]. These early investigations proposed that the MLA is a source of midfoot stiffness that makes our feet effective levers to push off from when walking and running [7,8]. However, subsequent investigations challenged this notion by providing evidence for considerable midfoot mobility [9–13]. Through this mobility, the MLA was also thought to increase locomotor economy by acting like a spring that stores and returns elastic energy with each step [14]. Given the debate around MLA stiffness and mobility, the transverse tarsal arch (TTA) of the foot has been proposed as an alternative source of significant longitudinal stiffness [15]. Other hypothesized adaptations in our feet, outside of their arches, include an enlarged calcaneal tuber in humans compared with predominantly quadrupedal African apes, which is seen as an adaptation to the larger forces encountered when striking the ground during bipedal gait [16]; and at the other end of the foot, shorter toes in humans, which are considered an adaptation to increase running economy [17].

Comparative studies such as those cited above are generally based on small numbers of feet assumed to be species-representative. However, human foot form has been extensively documented to exhibit great variability [18–20], which has led to the question whether variations in form are also related to variations in function *within* our species. Similar to the inter-species comparisons, several of the studies attempting to answer this question have focused on the MLA, the shape of which has commonly been represented simply by its height. A more expansive approach has been taken to characterize MLA function, using measures such as lower limb kinematics [21,22], ground reaction forces (GRFs) [23,24] and injury patterns [25–27]. This approach has led to conflicting results and a lack of consensus. Some studies claim MLA shape to be an important predictor of how someone walks and runs or the injuries they are at risk of sustaining [22,27], while others claim the contrary [21,23].

Rather than examining the complete morphology of the foot, most previous investigations have focused on specific foot components (e.g. calcaneal tuber length, toe length, MLA height) to represent its form. This approach might overlook important interactions that could offer further insights into the relationship between the many shape variations of our feet and overall mechanical function. Dissecting the foot into isolated components might also, as cautioned by Gould & Lewontin [28], result in looking for and proposing form–function relationships where none exists. Furthermore, Behling *et al*. [29], in their comprehensive review of the literature on human foot function to date, assert that evidence supporting theories of the foot acting as a mobile adaptor and rigid lever (centred around the MLA) is poor at best. Consequently, they, along with several others [30–32], advocate acknowledging the structural complexity and functional versatility of our feet. Hence, this study aims to evaluate foot form and function holistically, by constructing and analysing a statistical shape–function model (SFM) from three-dimensional (3D) foot scans and the ankle, subtalar, midtarsal, tarsometatarsal and metatarsophalangeal (MTP) joint angles and moments measured during treadmill walking and running with different inclinations. These joints capture the interactions between the established functional segments of the foot [33–35], and the locomotor tasks cover a wide range of energy requirements at the centre of mass to which the foot has been shown to contribute [36–38]. Furthermore, this study also aims to evaluate the accuracy with which the locomotor ankle and foot joint mechanics of a given foot can be predicted based solely on features of its external shape.

## 2. Methods

### (a) Participants

One hundred healthy adults, aged 18–40 years (50 females, 50 males; 25 ± 6 years; 175 ± 9 cm; 72 ± 12 kg), were recruited to participate in this study. Individuals with a lower limb injury 12 months prior, or any known neurological impairment, musculoskeletal or cardiovascular conditions were excluded from participating.

### (b) Experimental protocol and data analysis

#### (i) Foot morphology

The experimental protocol, data processing and analysis are described in detail in the supplementary material. Briefly, the participants' self-reported dominant foot was scanned using a FootIn3D scanner (Elinvision, Lithuania) while bearing minimal weight (mBW; approx. 10% bodyweight) and full bodyweight (fBW). All scans (regardless of weightbearing) were registered [39] and scaled to the same reference mesh. The resulting 3D Cartesian coordinates of the 29 528 vertices from each registered fBW mesh were used as the shape input for the SFM. The Euclidean vectors connecting corresponding vertices of mBW and fBW scans [40] were used as the deformation input for the SFM.

#### (ii) Foot function

To capture foot function during the selected locomotor tasks, participants were asked to walk and run barefoot (0.25 and 1.00 Froude number, respectively) at three inclinations (walking: −20, 0, 20%; running: −10, 0, 10%) on a force-sensing, fore–aft, tandem treadmill (AMTI, USA; 1000 Hz). A 14-camera 3D motion capture system (Qualisys, Sweden; 200 Hz) synchronously recorded marker [33] trajectories. From the resulting GRFs and body segment trajectories, foot and ankle joint angles and moments were determined using inverse kinematics and dynamics [35,41]. The stance time-normalized and step-averaged joint angle and moment curves for the ankle and foot joints of the dominant leg were used as the function input for the SFM.

### (c) Statistical analyses

To ensure a complete set of training data for constructing the SFM, joint angles and moments from missing uphill and downhill running trials were imputed for two participants [42,43].

**Figure 1.**
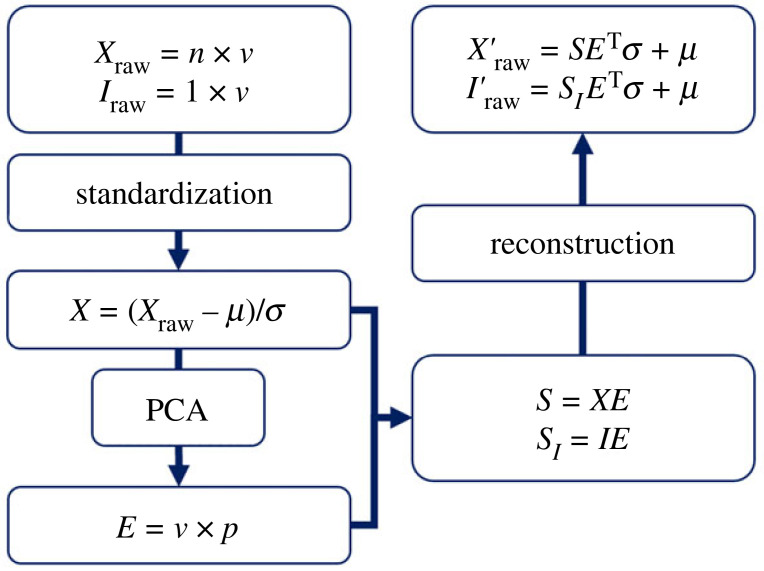
Schematic of the shape–function model construction process. The data for all *n* participants is stored in a matrix (*X*_raw_), where each row contains the *v* variables of foot shape, deformation and function for a single participant (*I*_raw_). Principal component analysis (PCA) is applied to the standardized input data matrix (*X*), which is centred around group means (*μ*) and scaled to group standard deviations (*σ*). The resulting eigenvector matrix (*E*) contains a transformation for each of the *v* variables and *p* principal components. The product of *X* and *E* is a matrix (*S* = *n* × *p*) that contains a principal component score for each participant. Accordingly, the transpose of the eigenvector matrix (*E*^T^), corresponding principal component scores for the entire sample (*S*) or a single participant (*S_I_*), *μ* and *σ* can be used to reconstruct the input data for all (*X’*_raw_) or a single participant (*I’*_raw_).

Figure 1 outlines the SFM construction process (following the methods described by Smoger *et al*. [44]) as well as how the resulting eigenvector matrix and principal component (PC) scores can be used to reconstruct the input data. For a detailed explanation, refer to the electronic supplementary material. The input data reconstruction was used to visualize the changes in shape, deformation and function described by the first three PCs, and to determine the accuracy of the SFM when predicting function from just shape, or from shape and deformation data. For the visualizations, the mean scores of each PC were perturbed by ±3 s.d. and then used to reconstruct the input variables.

To minimize the inherent subjectivity in interpreting PCs, the input data were reconstructed while emphasizing features captured by a particular PC by perturbing the scores for that PC. Two-sample *t*-tests and false discovery rate correction [45,46] were used to detect which reconstructed foot and deformation vertices experienced large displacements relative to their true counterparts. Colour mapping in the visualizations presented in figures 4–6 was used to accentuate the vertices that were identified as being displaced by more than 2 s.d. of all vertex displacements. Similarly, two-sample *t*-test statistical parametric mapping [47,48] was used to identify which regions of the various PC-perturbed and reconstructed joint angle and moment curves were displaced by more than 2 s.d. of all displacements. These regions are highlighted in figures 4–6 below. The electronic supplementary material contains a more detailed description of these tests.

**Figure 2.**
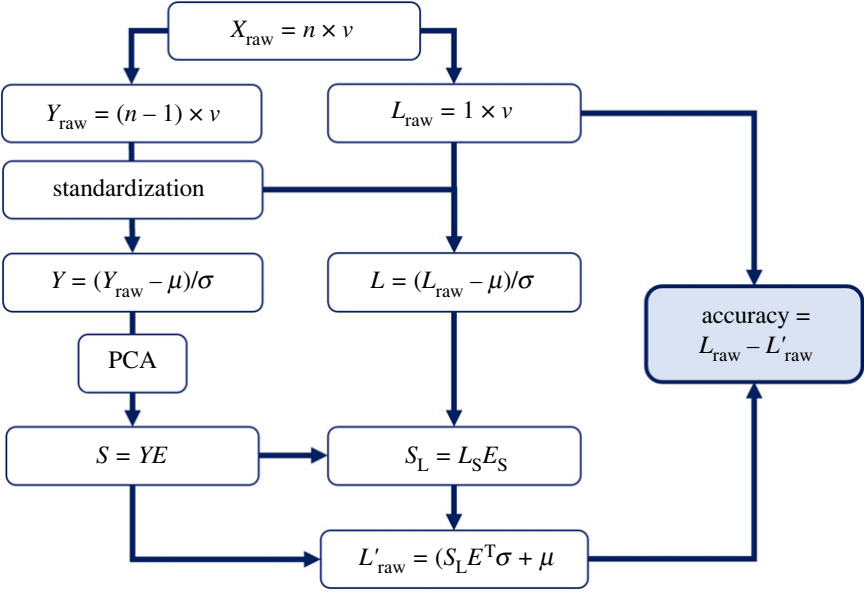
Schematic of the leave-one-out analysis to determine shape–function model accuracy. The *v* shape, deformation and function variables of a single participant (*L*_raw_) are first removed from the matrix of all *n* participants' data (*X*_raw_). Both the left-out participant's data and the data of all remaining participants (*Y*_raw_) are standardized by removing the means (*μ*) and scaling to the standard deviations (*σ*) of the remaining data. Principal component analysis (PCA) is performed on the standardized data (*Y*). The shape (or shape and deformation) portion of the resulting eigenvector matrix (*E*_s_) is combined with the shape (or shape and deformation) portion of the left-out participant's data (*L*_s_) to determine the principal component (PC) scores for the left-out participant (*S*_L_). Following the approach described by Smoger *et al*. [44], these PC scores and the entire eigenvector matrix (*E*) are then used to reconstruct the entire set of input data for the left-out participant (*L’*_raw_), which is compared with the true data.

Foot function prediction accuracy was assessed using a leave-one-out (LOO) analysis. This analysis involved iteratively repeating the process described in figure 2 while omitting a different participant from the input data matrix. The accuracy of predicted foot function is reported as the sample mean agreement between the true and predicted time-series using the strength of fit (*R*^2^), slope and intercept of linear regression models [11,49] and root mean square distance (RMSD) across the entire stance phase for each joint, during each locomotor task.

**Figure 3.**
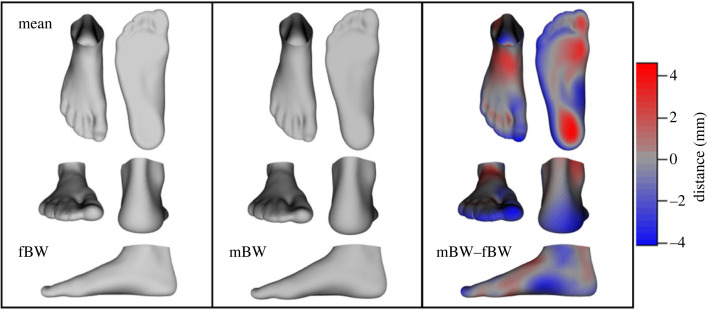
Mean foot shape while bearing full bodyweight (fBW) and minimal weight (mBW), as well as mean deformations when increasing the load from mBW to fBW (mBW–fBW). The colour coding on the mean deformed foot depicts the areas and magnitude of differences between the mean mBW foot and the mean deformed foot.

## 3. Results

### (a) Statistical foot shape–function model

**Figure 4.**
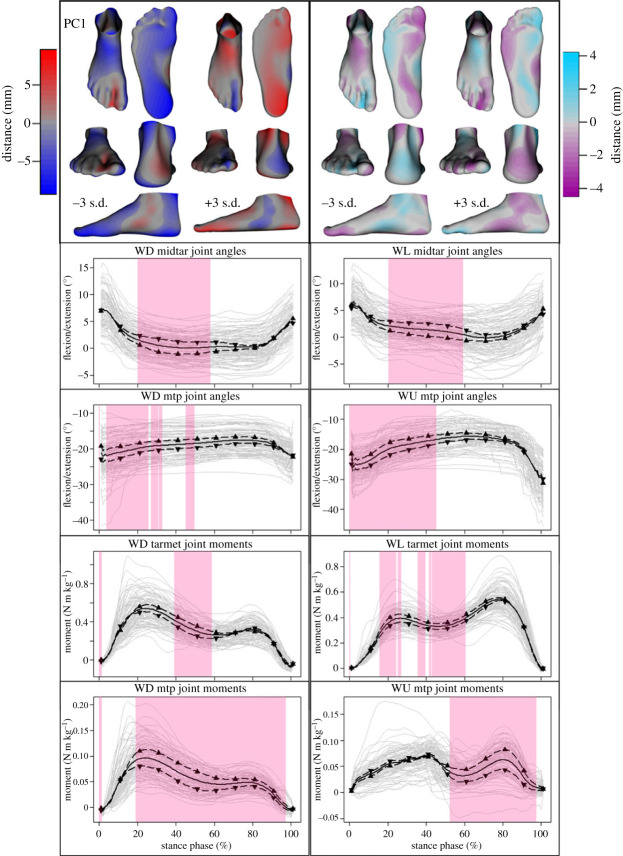
Mean ± 3 s.d. of shape (top left), deformation (top right), and joint angle and moment variations described by the first principal component (PC1). Colour coding depicts the degree of deviation from the mean foot or mean deformed foot (figure 3). Midtarsal (midtar) and metatarsophalangeal (mtp) joint angles while walking downhill (WD), level (WL) and uphill (WU) are shown. Joint moments of the tarsometatarsal (tarmet) joint during WD and WL, as well as of the mtp joint WD and WU are shown. Upward-pointing triangles denote +3 s.d. and downward-pointing triangles −3 s.d. Mean (solid black line) and participant means (grey lines) are also included as a reference. The highlighted regions signify where statistical parametric mapping identified substantial differences in the time-series.

**Figure 5.**
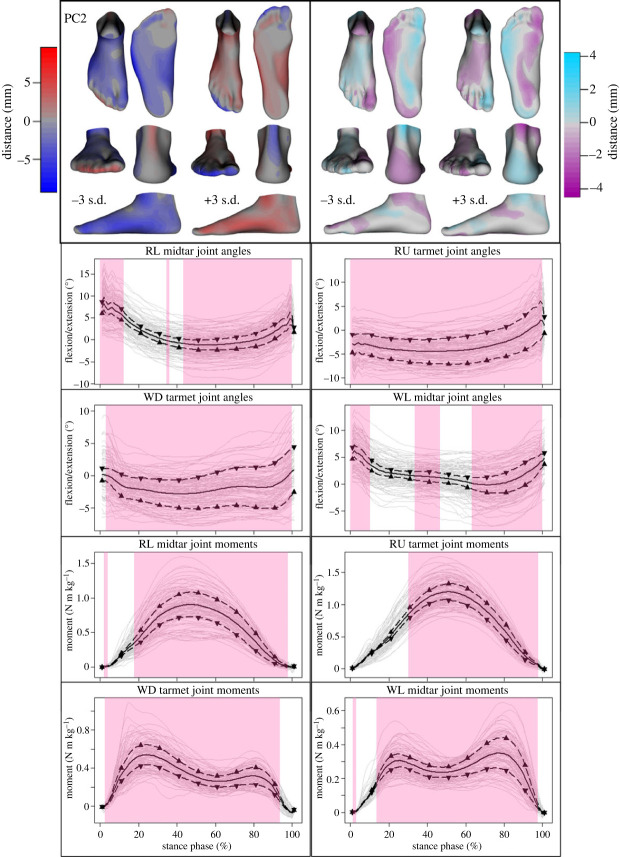
Mean ± 3 s.d. of shape (top left), deformation (top right), and joint angle and moment variations described by the second principal component (PC2). Colour coding depicts the degree of deviation from the mean foot or mean deformed foot (figure 3). Midtarsal (midtar) and tarsometatarsal (tarmet) joint angles and moments during level (RL) and uphill running (RU), as well as level (WL) and downhill walking (WD) are shown. Upward-pointing triangles denote +3 s.d. and downward-pointing triangles −3 s.d. Mean (solid black line) and participant means (grey lines) are also included as a reference. The highlighted regions signify where statistical parametric mapping identified substantial differences in the time-series.

**Figure 6.**
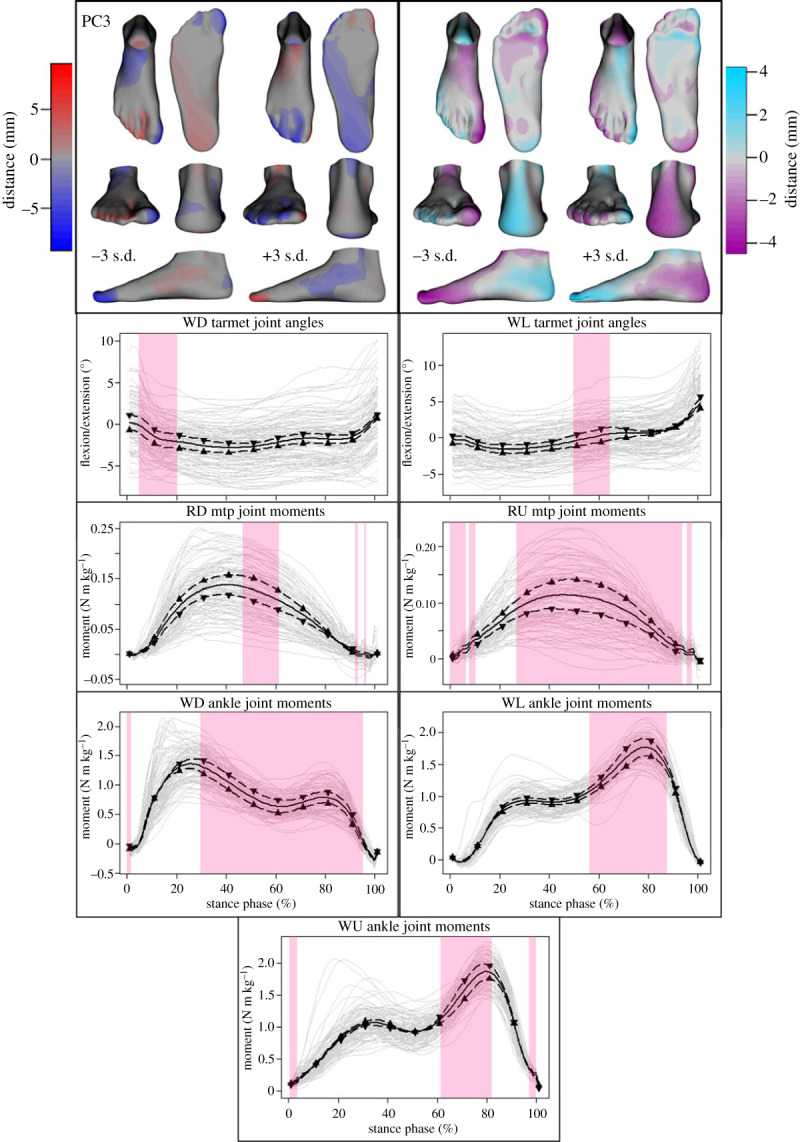
Mean ± 3 s.d. of shape (top left), deformation (top right), and joint angle and moment variations described by the third principal component (PC3). Colour coding depicts the degree of deviation from the mean foot or mean deformed foot (figure 3). Tarsometatarsal (tarmet) joint angles during downhill (WD) and level walking (WL) are shown. Joint moments of the metatarsophalangeal (mtp) joint during uphill (RU) and downhill running (RD), and ankle joint during WD, WL and uphill walking (WU) are shown. Upward-pointing triangles denote +3 s.d. and downward-pointing triangles −3 s.d. Mean (solid black line) and participant means (grey lines) are also included as a reference. The highlighted regions signify where statistical parametric mapping identified substantial differences in the time-series.

The SFM reduced 183 228 foot shape, deformation and function inputs to 100 PCs. The first eight PCs captured 53.1% of the variability contained within the entire dataset, while 21, 34, 50 and 100 PCs explained 75.3, 85.1, 91.7 and 100%, respectively. To help understand the relationships between foot form and function identified by the first three PCs, the changes in shape, deformation and function resulting from perturbations to them are presented in figures 4–6, as well as in figures and animations in the electronic supplementary material.

The first PC explained 14.7% of the total variability and captured shape variations that include differences in MLA height, heel and forefoot width and toe splay. The associated differences in deformation due to increased loading included MLA compression, ankle internal/external rotation and medial/lateral plantar compression. Specifically, supinated feet with a high MLA and wide forefoot (−3 s.d.) exhibited ankle external rotation and limited MLA compression, but with substantial forefoot splay. These feet also appeared to cover a smaller midtarsal joint range of motion (ROM) throughout stance than their pronated, low-arched counterparts (+3 s.d.), while exhibiting greater MTP joint dorsiflexion throughout the first 50% of stance of the different walking tasks. High-arched feet also displayed lower tarsometatarsal and MTP joint moments during walking midstance (approx. 30–60%) and throughout most of the stance phase (approx. 20–95%), respectively (figure 4).

The second PC accounted for 8.6% of the total variability and described variations in overall foot width, relative lengths of the forefoot, rearfoot and lesser toes, and TTA curvature. The covarying deformations showed that a wide foot with reduced TTA curvature and short forefoot and toes (−3 s.d.) was likely to evert at the ankle and forefoot when loaded. In contrast, a narrow foot with high TTA curvature and long forefoot and toes (+3 s.d.) was likely to invert at the ankle and forefoot. The former also exhibited greater midtarsal plantarflexion during the first 10 and last 40–50% of stance, and tarsometatarsal plantarflexion and ROM throughout stance. Simultaneously, these feet exhibited smaller moments throughout most of the stance phase for both these joints, as well as the ankle, subtalar and MTP joints, regardless of locomotor task (figure 5).

Accounting for 7.1% of the total variability, the third PC described variations in the orientation of the forefoot relative to the rearfoot, size and angle of the hallux, as well as the height and width of the calcaneal tuberosity. Feet with a large, straight hallux, adducted forefoot and tall heel (−3 s.d.) displayed less rearfoot abduction, and less hallux but more lesser-toe flexion when loaded. These feet also exhibited greater tarsometatarsal plantarflexion during short periods of the stance phase, alongside higher ankle joint moments during mid to late stance in all walking tasks. PC3 also described differences in MTP joint moments. However, in this instance, feet with a smaller adducted hallux that displayed increased hallux compression during loading (+3 s.d.) also experienced greater MTP joint moments during uphill and downhill running (figure 6).

### (b) Predicting foot function from shape

The LOO analysis revealed moderate–excellent agreement between measured and predicted joint angles and moments, regardless of joint or locomotor task. The supplementary material contain detailed results for predictions of joint angles and moments using only foot shape and shape and deformation characteristics during the various locomotor tasks. In general, mean *R*^2^ values were more than or equal to 0.5, while mean slopes ranged from 0.7 to 1.0. Although these mean values indicate moderate–excellent agreement, it is worth noting that the ranges show no or poor agreement in some participants for some joints and locomotor tasks (e.g. midtarsal, subtalar and tarsometatarsal joint angles during level walking).

**Figure 7.**
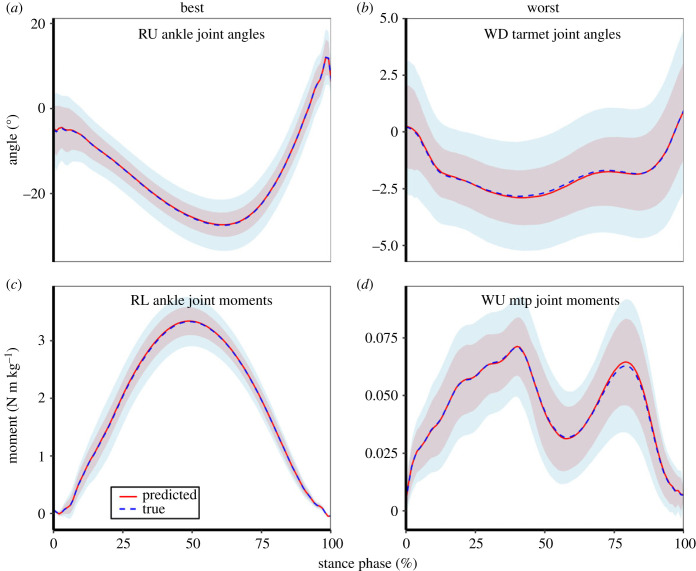
Time-normalized measured (true) and leave-one-out predicted joint angles (*a*,*b*) and moments (*c*,*d*) for the joints and locomotor tasks with the highest (best; *a*,*c*) and lowest (worst; *b*,*d*) agreement using only shape characteristics. The ankle joint predictions produced the best agreement for joint angles during uphill running (RU) and joint moments during level running (RL). The tarsometatarsal (tarmet) joint produced the lowest level of agreement between predicted and measured joint angles during downhill walking (WD), while the metatarsophalangeal (mtp) joint produced the lowest level of agreement between predicted and measured joint moments during uphill walking (WU). Time-series are presented as means ± 1 s.d. of the entire sample.

When using only foot shape characteristics to predict function, the best agreements were found for joint angles of the ankle during uphill running (*R*^2^ = 0.949, slope = 0.994, intercept = −0.082°, RMSD = 5.615°) and joint moments of the same joint during level running (*R*^2^ = 0.979, slope = 1.000, intercept = 0.002 N m kg^−1^, RMSD = 0.317 N m kg^−1^). The worst agreements were found for tarsometatarsal joint angles during downhill walking (*R*^2^ = 0.495, slope = 0.877, intercept = −0.262°, RMSD = 2.288°) and MTP joint moments during uphill walking (*R*^2^ = 0.600, slope = 0.899, intercept = 0.005 N m kg^−1^, RMSD = 0.020 N m kg^−1^). Figure 7, which displays the true and predicted joint angle and moment time-series of the joints and locomotor tasks with the best and worst agreement, provides context to these results.

Using both shape and deformation data to predict joint angles and moments resulted in similar degrees of agreement between true and predicted time-series. Ankle joint angles during uphill running (*R*^2^ = 0.934, slope = 0.987, intercept = −0.270°, RMSD = 5.641°) and ankle joint moments during level running (*R*^2^ = 0.975, slope = 0.994, intercept = 0.008 N m kg^−1^, RMSD = 0.330 N m kg^−1^) displayed the greatest agreement. In contrast, tarsometatarsal joint angles during downhill walking (*R*^2^ = 0.499, slope = 0.702, intercept = −0.523°, RMSD = 2.171°) and MTP joint moments during uphill walking (*R*^2^ = 0.551, slope = 0.808, intercept = 0.009 N m kg^−1^, RMSD = 0.022 N m kg^−1^) showed the least agreement. The accuracy for both predicted joint angles and moments decreased slightly compared with shape-only predictions. Agreement for predicted joint angles fell from a range of mean *R*^2^ values of 0.495–0.949 to 0.499–0.934. Agreement for predicted joint moments decreased from 0.600–0.979 to 0.551–0.975.

## 4. Discussion

The principal component analysis (PCA)-based statistical shape–function modelling approach taken in this study did not require *a priori* selection of specific predictor and dependent variables. This allows the interpretation of all identified relationships in the context of the foot as a whole, while avoiding the use of simplified representations of the complex form and function of the foot. An additional advantage of this approach is that the information contained within the SFM can be used to predict the kinematics and kinetics of healthy feet (novel to the training dataset) with moderate–excellent accuracy, based on both foot shape only and the combination of shape and deformation. Together, the insight provided by this foot SFM and its predictive capacity have the potential to impact a wide range of fields, including evolutionary anthropology, musculoskeletal and orthopaedic medicine, and shoe design.

### (a) The medial longitudinal and transverse tarsal arches

Though the results from this investigation support some of the previous research around the MLA, they also provide important context relative to the many other components of the foot. Like the first PC in prior statistical foot shape models [19,40,50], the current SFM primarily described MLA shape variations, underscoring the magnitude of variation therein. Moreover, both the covariations in load-induced shape deformations, and the midtarsal joint kinematics during locomotion support the notion that the MLA of a high-arched foot is less likely to compress than that of a low-arched foot, under both static [51–54] and dynamic loading [55–57]. Nonetheless, this first PC accounted for only approximately 15% of the total variability, which indicates that the mechanical characteristics of a given foot cannot be defined based solely on the shape of its MLA.

Transverse tarsal arch curvature covaried with increased midfoot moments and reduced tarsometatarsal ROM (PC2), which suggests increased TTA curvature is associated with increased MLA stiffness. To our knowledge, this is the first *in vivo*, locomotion-based evidence for the relationship between TTA curvature and MLA stiffness, as proposed by Venkadesan *et al*. [15]. Importantly, this finding highlights that foot stiffness is influenced by both MLA and TTA shape, despite the shapes of the MLA and TTA appearing to be independent of each other (described in different PCs). This further highlights the multi-factorial nature of foot stiffness and the complex and variable nature of human foot morphology.

### (b) Relative proportions and orientation of forefoot to rearfoot

Ankle, subtalar, midtarsal, tarsometatarsal and MTP joint moments were all associated with the relative proportions of the foot, as described in PC2. Larger ankle, subtalar, midfoot and MTP joint moments coincide with a long forefoot, short rearfoot and narrow foot shape (+3 s.d.). This link can be explained by the fact that, as relative foot width decreases and relative forefoot length increases, so does the moment arm between the respective GRF components’ point of application and the joints' centre of rotation.

Contrary to our findings on foot proportions, previous investigations have reported increased computer-simulated plantar flexor work as a consequence of an increased forefoot to rearfoot length ratio [58], an inferred increase in elastic energy utilization in the Achilles tendon associated with a shorter calcaneal tuber length [59,60], and increased elastic energy reutilization in the ankle spring of a prosthesis with longer heel-to-toe-joint length [61]. These investigations all associated foot proportions to ankle mechanics during running, but the relationship between calcaneal tuber length and running economy (from which elastic energy utilization in the Achilles tendon was inferred) did not translate to walking [59,62], contrary to the results presented here. The discrepancy in the previously reported link between relative foot proportions and ankle mechanics, and that described by PC2 here could be due to the former using simulated or inferred ankle mechanics, whereas our SFM uses *in vivo* ankle joint angles and moments. Moreover, PC2 does not exclusively describe the ratio of forefoot to rearfoot length, but also describes differences in the overall width of the foot and relative length of the lesser toes, both of which might also affect ankle mechanics during the various locomotor tasks.

### (c) Hallux and lesser-toe lengths and orientation

Further notable differences between PC2 and 3 lie in the variations in toe length and MTP joint moments. Only the hallux varies in length in PC3, whereas in PC2, the length of the lesser toes varies. While some investigations have found that neither orientation nor length (absolute or relative) of the toes affects walking dynamics [61,63], others have found that toe length does affect running performance [17,64,65]. Two of these investigations suggest that longer toes (especially a longer second toe) create larger MTP joint moments, which in turn affects plantar flexor gearing in such a way that benefits both sprinting [64] and endurance running [65]. Correspondingly, in our SFM, variations in hallux length are associated with some variations in MTP joint moments during uphill and downhill running (PC3), whereas variations in lesser-toe lengths are associated with large variations in MTP joint angles regardless of locomotor task (PC2).

### (d) Predicting foot function from shape

The external shape of a given foot can generally be used to predict its mechanical function. However, for some feet, the current SFM is unable to accurately predict the mechanics of certain joints during certain locomotor tasks (e.g. figure of best and worst individual predictions in the electronic supplementary material). This inability to accurately predict joint mechanics can be attributed to two possible explanations. Firstly, the SFM does not generalize well, meaning that the breadth of information contained in the SFM is insufficient to accurately represent certain unknown instances. While this highlights the variability in joint mechanics across the population, previously shown by Nester *et al*. [66] using a less constrained musculoskeletal foot model but in a comparable sample of 100 healthy adults, it also shows that large datasets (*N* > 99) are required to accurately represent this variability.

Secondly, certain variations in ankle and foot joint mechanics lack strong covariates of external foot shape or deformation. In other words, two feet with very similar shape and deformation characteristics may nevertheless display very different ankle and foot joint mechanics during locomotion, in line with McClymont, Davids & Crompton's proposition of neurobiological degeneracy [31]. Both explanations, together with the high intra-individual variability shown for spatio-temporal gait parameters [67] and mean and maximum plantar pressures [68], emphasize the need for large datasets to accurately represent complex systems such as our feet.

### (e) General implications of the statistical shape–function model

Above all else, the results from this investigation highlight the complexity and high variability of the foot, in both form and function. The largest PC, as well as the following seven required to explain just over half of the total variability, all account for relatively small proportions of the total variability (i.e. small eigenvalues). This shows that, owing to the foot's structural complexity, the relationships between form and function may not be tightly constrained. In comparison, SFMs of less complex biological structures like the knee have accounted for over 45% of the variability in shape and function with just the first PC [44,69]. Moreover, the small relative weight of the specific shape–function relationships described by each PC is comparable with previous reports of weak-to-moderate relationships between the shape and function characteristics of specific foot structures [21,52,70,71]. However, our SFM is the first to simultaneously consider all the previously described shape–function relationships and assign them a hierarchical order of importance. It thereby confirms the relative significance and large variability of the MLA, while also emphasizing the importance of foot proportions, and toe orientation and length.

The small eigenvalues also imply that the large degrees of freedom afforded by the structural complexity of the foot allow multiple means of achieving the same functional outcome. That is, despite large variations in foot shape and mechanics, all participants were able to successfully perform each of the locomotor tasks. As Lauder [72, p. 1] argues, function is not defined by musculoskeletal structure itself, but by how it is used, i.e. ‘the motor programs in the central nervous system’. There is ample evidence for this argument in relation to the foot. Various investigations have shown that the neuromuscular control of intrinsic and extrinsic foot muscles plays an important role in the mechanical function and versatility of the foot [36–38,73]. Thus, future investigations examining the link between foot form and function might benefit from also examining the potential link to the neuromuscular control of relevant muscles as well as afferent feedback from proprioceptive and cutaneous receptors during various locomotor tasks. Furthermore, Lauder [72] points out that the definition of function can vary widely. In the current investigation, it was defined as the joint kinematics and kinetics produced to complete locomotor tasks with varying mechanical requirements. However, many other investigations have defined or compared function in terms of the metabolic cost of locomotion [59,62,74] or performance during a locomotor task [64,65]. Since these definitions of function have greater implications for general human behaviour (considering we intuitively minimize energy expenditure [75]), linking foot form and mechanics to locomotor economy using a similar approach to that used here might help resolve some of the remaining questions regarding the link between foot structure and locomotor economy. Such an approach could help resolve whether there are also variations in anatomical parameters that are as closely related to walking economy [62] as heel length is to running economy [59], or whether short [17] or long toes [65] are better for endurance running.

The range of variation in foot form and function, the relationships between them and the predictive capacity of the SFM all have applications across a broad range of fields. The wide variability in foot shape and function contained within the SFM could be used as a comparison against the shapes and functions inferred from fossil foot bones and footprints, for insights into how modern human foot function relates to the patterns that may have characterized our extinct ancestors and relatives. The various form–function relationships the SMF describes could also be used to inform the design of shoes aimed at enhancing running performance, since the impact shoe design can have on performance has been found to vary considerably across runners [76]. Based on the aforementioned form–function relationships, a low-arched foot might benefit from increased midsole bending stiffness, whereas a foot with a relatively long heel and short forefoot might benefit from a compliant midsole. Thus, it might be possible to identify the ideal shoe for a particular runner based on their foot shape characteristics. Lastly, if the complete external shape of fossilized feet and footprints can be reconstructed from partial data, as has been done for skeletal foot segments [77], the predictive capacity of the SFM could provide a more complete understanding of their inferred mechanical function. Similarly, inferring mechanical function from shape could serve as a diagnostic tool for podiatrists and orthopaedic surgeons, providing insight through the comparison of predicted healthy mechanics and those measured from an impaired foot.

### (f) Limitations

The findings of this study must be considered in light of some limitations. Firstly, a common limitation of studies using a PCA-based approach to identify the major modes of variation or covariation in a set of complex data is that the interpretation of the resulting PCs can be somewhat subjective. To address this limitation, we used statistical tests to highlight regions of major variation. Animations (included in the electronic supplementary material) were used to further emphasize the areas and nature of shape changes. To further reduce subjectivity in future investigations, it may be useful to correlate common measures of shape characteristics (e.g. foot length, width or arch index obtained from 3D foot scans) to their respective PC scores, as Smoger *et al*. [44] have done. Another limitation to consider regarding the foot function data is that only the kinematics and kinetics during the stance phase were analysed. Considering the entire stride along with additional factors such as spatio-temporal gait variables (stride length and frequency) might also account for some of the variation in kinematics and kinetics. Lastly, since our sample consisted of only young, healthy participants, the results presented here may not be transferable to clinical, juvenile or elderly populations. And, while we tried to cover a diverse range of locomotor tasks with different energetic requirements, human movement is far more diverse (e.g. changing direction, jumping, climbing, etc.), as a result of which the tasks chosen may not fully encapsulate the functional capacities (and their variations) of modern human feet.

## 5. Conclusion

Using a PCA-based approach to identify relationships between external foot shape, deformations and joint mechanics during locomotion has revealed that, across a healthy, young population, the greatest variability exists in the MLA, TTA, relative foot proportions and toe shape characteristics as well as their associated deformations and joint mechanics. While the relationships between these various aspects of external foot shape and internal joint mechanics agree with similar findings from previous research, context regarding their relevance to overall foot form and function is provided for the first time to our knowledge by the relatively small proportion of overall variability accounted for by each of these covariations. Beyond describing the various links between shape characteristics and joint mechanics, the foot SFM presented here has potential applicability across a wide range of fields, as it can predict the internal joint mechanics of a given foot with moderate–excellent accuracy based only on its external shape and deformations.

## Data Availability

The 3D foot scans (PLY files), and locomotor foot and ankle joint angles and moments (MATLAB structures) can be downloaded from Dryad: https://doi.org/10.5061/dryad.g4f4qrfwt [78]. Some results are provided in electronic supplementary material [79].

## References

[1] Gould SJ. 2002 The structure of evolutionary theory. Cambridge, MA: Harvard University Press.

[2] Darwin C, Wallace A. 1858 On the variation of organic beings in a state of nature. J. Proc. Linn. Soc. Lond. **3**, 45-52.

[3] Huxley J. 1942 Evolution. The modern synthesis. London, UK: George Allen & Unwin.

[4] Hunt KD. 2015 Bipedalism. In Basics in human evolution (ed. MP Muehlenbein), pp. 103-112. Boston, MA: Academic Press.

[5] Bramble DM, Lieberman DE. 2004 Endurance running and the evolution of *Homo*. Nature **432**, 345-352. (10.1038/nature03052) 15549097

[6] Morton DJ. 1924 Evolution of the longitudinal arch of the human foot. J. Bone Joint Surg. **6**, 56-90.

[7] Hicks JH. 1955 The foot as a support. Cells Tissues Organs **25**, 34-45. (10.1159/000141055)

[8] Elftman H, Manter J. 1935 Chimpanzee and human feet in bipedal walking. Am. J. Phys. Anthropol. **20**, 69-79. (10.1002/ajpa.1330200109)

[9] Welte LKM, Kelly LA, Kessler SE, Lieberman DE, D'Andrea SE, Lichtwark GA, Rainbow MJ. 2021 The extensibility of the plantar fascia influences the windlass mechanism during human running. Proc. R. Soc. B **288**, 20202095. (10.1098/rspb.2020.2095) PMC7893268 33468002

[10] Welte LKM, Kelly LA, Lichtwark GA, Rainbow MJ. 2018 Influence of the windlass mechanism on arch-spring mechanics during dynamic foot arch deformation. J. R. Soc. Interface **15**, 20180270. (10.1098/rsif.2018.0270) 30111662 PMC6127178

[11] Kessler SE, Rainbow MJ, Lichtwark GA, Cresswell AG, D'Andrea SE, Konow N, Kelly LA. 2019 A direct comparison of biplanar videoradiography and optical motion capture for foot and ankle kinematics. Front. Bioeng. Biotechnol. **7**, 199. (10.3389/fbioe.2019.00199) 31508415 PMC6716496

[12] Holowka NB, O'Neill MC, Thompson NE, Demes B. 2017 Chimpanzee and human midfoot motion during bipedal walking and the evolution of the longitudinal arch of the foot. J. Hum. Evol. **104**, 23-31. (10.1016/j.jhevol.2016.12.002) 28317554

[13] Lundgren P, Nester CJ, Liu A, Arndt A, Jones R, Stacoff A, Wolf P, Lundberg A. 2008 Invasive *in vivo* measurement of rear-, mid- and forefoot motion during walking. Gait Posture **28**, 93-100. (10.1016/j.gaitpost.2007.10.009) 18096389

[14] Ker RF, Bennett MB, Bibby SR, Kester RC, Alexander RM. 1987 The spring in the arch of the human foot. Nature **325**, 147-149. (10.1038/325147a0) 3808070

[15] Venkadesan M, Yawar A, Eng CM, Dias MA, Singh DK, Tommasini SM, Haims AH, Bandi MM, Mandre S. 2020 Stiffness of the human foot and evolution of the transverse arch. Nature **579**, 97-100. (10.1038/s41586-020-2053-y) 32103182

[16] Latimer BM, Lovejoy CO. 1989 The calcaneus of *Australopithecus afarensis* and its implications for the evolution of bipedality. Am. J. Phys. Anthropol. **78**, 369-386. (10.1002/ajpa.1330780306) 2929741

[17] Rolian C, Lieberman DE, Hamill J, Scott JW, Werbel W. 2009 Walking, running and the evolution of short toes in humans. J. Exp. Biol. **212**, 713-721. (10.1242/jeb.019885) 19218523

[18] Cavanagh PR, Rodgers MM. 1987 The arch index: a useful measure from footprints. J. Biomech. **20**, 547-551. (10.1016/0021-9290(87)90255-7) 3611129

[19] Stanković K, Booth BG, Danckaers F, Burg F, Vermaelen P, Duerinck S, Sijbers J, Huysmans T. 2018 Three-dimensional quantitative analysis of healthy foot shape: a proof of concept study. J. Foot Ankle Res. **11**, 8. (10.1186/s13047-018-0251-8) 29541162 PMC5845135

[20] Domjanic J, Seidler H, Mitteroecker P. 2015 A combined morphometric analysis of foot form and its association with sex, stature, and body mass. Am. J. Phys. Anthropol. **157**, 582-591. (10.1002/ajpa.22752) 25846015

[21] Nigg BM, Cole GK, Nachbauer W. 1993 Effects of arch height of the foot on angular motion of the lower extremities in running. J. Biomech. **26**, 909-916. (10.1016/0021-9290(93)90053-H) 8349716

[22] Williams III DS, McClay IS, Hamill J, Buchanan TS. 2001 Lower extremity kinematic and kinetic differences in runners with high and low arches. J. Appl. Biomech. **17**, 153. (10.1123/jab.17.2.153)

[23] Nachbauer W, Nigg BM. 1992 Effects of arch height of the foot on ground reaction forces in running. Med. Sci. Sports Exercise **24**, 1264-1269. (10.1249/00005768-199211000-00011) 1359377

[24] Williams III DS, Tierney RN, Butler RJ. 2014 Increased medial longitudinal arch mobility, lower extremity kinematics, and ground reaction forces in high-arched runners. J. Athl. Train. **49**, 290-296. (10.4085/1062-6050-49.3.05) 24840580 PMC4080592

[25] Hagedorn TJ, Dufour AB, Riskowski JL, Hillstrom HJ, Menz HB, Casey VA, Hannan MT. 2013 Foot disorders, foot posture, and foot function: the Framingham foot study. PLoS ONE **8**, e74364. (10.1371/journal.pone.0074364) 24040231 PMC3764219

[26] Riskowski JL, Dufour AB, Hagedorn TJ, Hillstrom HJ, Casey VA, Hannan MT. 2013 Associations of foot posture and function to lower extremity pain: results from a population-based foot study. Arthrit. Care Res. **65**, 1804-1812. (10.1002/acr.22049) PMC4039193 24591410

[27] Tong JWK, Kong PW. 2013 Association between foot type and lower extremity injuries: systematic literature review with meta-analysis. J. Orthopaed. Sports Phys. Therapy **43**, 700-714. (10.2519/jospt.2013.4225) 23756327

[28] Gould SJ, Lewontin RC. 1979 The spandrels of San Marco and the Panglossian paradigm: a critique of the adaptationist programme. Proc. R. Soc. Lond. B **205**, 581-598. (10.1098/rspb.1979.0086) 42062

[29] Behling A-V, Rainbow MJ, Welte LKM, Kelly LA. 2023 Chasing footprints in time – reframing our understanding of human foot function in the context of current evidence and emerging insights. Biol. Rev. **98**, 2136-2151, (10.1111/brv.12999) 37489055

[30] Hatala KG, Gatesy SM, Falkingham PL. 2023 Arched footprints preserve the motions of fossil hominin feet. Nat. Ecol. Evol. **7**, 32-41. (10.1038/s41559-022-01929-2) 36604550

[31] McClymont J, Davids K, Crompton RH. 2022 Variation, mosaicism and degeneracy in the hominin foot. Evol. Hum. Sci. **4**, e2. (10.1017/ehs.2021.50) 37588898 PMC10426032

[32] DeSilva JM, Bonne-Annee R, Swanson Z, Gill CM, Sobel M, Uy J, Gill SV. 2015 Midtarsal break variation in modern humans: functional causes, skeletal correlates, and paleontological implications. Am. J. Phys. Anthropol. **156**, 543-552. (10.1002/ajpa.22699) 25594359

[33] Leardini A, Benedetti MG, Berti L, Bettinelli D, Nativo R, Giannini S. 2007 Rear-foot, mid-foot and fore-foot motion during the stance phase of gait. Gait Posture **25**, 453-462. (10.1016/j.gaitpost.2006.05.017) 16965916

[34] Malaquias TM, Silveira C, Aerts W, De Groote F, Dereymaeker G, Vander Sloten J, Jonkers I. 2017 Extended foot-ankle musculoskeletal models for application in movement analysis. Comput. Methods Biomech. Biomed. Eng. **20**, 153-159. (10.1080/10255842.2016.1206533) 27381808

[35] Maharaj JN, Rainbow MJ, Cresswell AG, Kessler SE, Konow N, Gehring D, Lichtwark GA. 2021 Modelling the complexity of the foot and ankle during human locomotion: the development and validation of a multi-segment foot model using biplanar videoradiography. Comput. Methods Biomech. Biomed. Eng. **25**, 554-565. (10.1080/10255842.2021.1968844) 34698598

[36] Birch JV, Kelly LA, Cresswell AG, Dixon SJ, Farris DJ. 2021 Neuromechanical adaptations of foot function to changes in surface stiffness during hopping. J. Appl. Physiol. **130**, 1196-1204. (10.1152/japplphysiol.00401.2020) 33571058

[37] Riddick R, Farris DJ, Kelly LA. 2019 The foot is more than a spring: human foot muscles perform work to adapt to the energetic requirements of locomotion. J. R. Soc. Interface **16**, 20180680. (10.1098/rsif.2018.0680) 30958152 PMC6364639

[38] Smith RE, Lichtwark GA, Kelly LA. 2021 The energetic function of the human foot and its muscles during accelerations and decelerations. J. Exp. Biol. **224**, jeb242263. (10.1242/jeb.242263) 34018550

[39] Danckaers F, Huysmans T, Lacko D, Ledda A, Verwulgent S, Van Dongen S, Sijbers J. 2014 Correspondence preserving elastic surface registration with shape model prior. In *Proc. 22nd Int. Conf. Pattern Recognition, Stockholm, Sweden, 24–28 August 2014*, pp. 2143–2148. Piscataway, NJ: IEEE. (10.1109/ICPR.2014.373)

[40] Schuster RW, Cresswell AG, Kelly LA. 2021 Reliability and quality of statistical shape and deformation models constructed from optical foot scans. J. Biomech. **115**, 110137. (10.1016/j.jbiomech.2020.110137) 33267964

[41] Delp SL, Anderson FC, Arnold AS, Loan P, Habib A, John CT, Guendelman E, Thelen DG. 2007 OpenSim: open-source software to create and analyze dynamic simulations of movement. IEEE Trans. Biomed. Eng. **54**, 1940-1950. (10.1109/TBME.2007.901024) 18018689

[42] Dray S, Josse J. 2015 Principal component analysis with missing values: a comparative survey of methods. Plant Ecol. **216**, 657-667. (10.1007/s11258-014-0406-z)

[43] Josse J, Husson F. 2016 missMDA: a package for handling missing values in multivariate data analysis. J. Stat. Softw. **70**, 1-31. (10.18637/jss.v070.i01)

[44] Smoger LM, Fitzpatrick CK, Clary CW, Cyr AJ, Maletsky LP, Rullkoetter PJ, Laz PJ. 2015 Statistical modeling to characterize relationships between knee anatomy and kinematics. J. Orthop. Res. **33**, 1620-1630. (10.1002/jor.22948) 25991502 PMC4591110

[45] Stanković K, Huysmans T, Danckaers F, Sijbers J, Booth BG. 2020 Subject-specific identification of three dimensional foot shape deviations using statistical shape analysis. Expert Systems Appl. **151**, 113372. (10.1016/j.eswa.2020.113372)

[46] Storey JD. 2011 False discovery rate. Int. Encycl. Stat. Sci. **1**, 504-508. (10.1007/978-3-642-04898-2_248)

[47] Pataky TC. 2011 One-dimensional statistical parametric mapping in Python. Comput. Methods Biomech. Biomed. Eng. **15**, 295-301. (10.1080/10255842.2010.527837) 21756121

[48] Pataky TC, Robinson M, Vanrenterghem J. 2013 Vector field statistical analysis of kinematic and force trajectories. J. Biomech. **46**, 2394-2401. (10.1016/j.jbiomech.2013.07.031) 23948374

[49] Iosa M, Cereatti A, Merlo A, Campanini I, Paolucci S, Cappozzo A. 2014 Assessment of waveform similarity in clinical gait data: the linear fit method. BioMed Res. Int. **2014**, 214156. (10.1155/2014/214156) 25126548 PMC4122015

[50] Schuster RW, Cresswell AG, Kelly LA. 2023 Foot shape is related to load-induced shape deformations, but neither are good predictors of plantar soft tissue stiffness. J. R. Soc. Interface **20**, 20220758. (10.1098/rsif.2022.0758) 36651181 PMC9846431

[51] Arangio GA, Chen C, Salathé EP. 1998 Effect of varying arch height with and without the plantar fascia on the mechanical properties of the foot. Foot Ankle Int. **19**, 705-709. (10.1177/107110079801901010) 9801086

[52] Zifchock RA, Davis I, Hillstrom HJ, Song J. 2006 The effect of gender, age, and lateral dominance on arch height and arch stiffness. Foot Ankle Int. **27**, 367-372. (10.1177/107110070602700509) 16701058

[53] Zifchock RA, Theriot C, Hillstrom HJ, Song J, Neary M. 2017 The relationship between arch height and arch flexibility: a proposed arch flexibility classification system for the description of multidimensional foot structure. J. Am. Podiatr. Med. Assoc. **107**, 119-123. (10.7547/15-051) 28198638

[54] Cornwall MW, McPoil TG. 2011 Relationship between static foot posture and foot mobility. J. Foot Ankle Res. **4**, 4. (10.1186/1757-1146-4-4) 21244705 PMC3033808

[55] Buldt AK, Levinger P, Murley GS, Menz HB, Nester CJ, Landorf KB. 2015 Foot posture is associated with kinematics of the foot during gait: a comparison of normal, planus and cavus feet. Gait Posture **42**, 42-48. (10.1016/j.gaitpost.2015.03.004) 25819716

[56] Kruger KM, Graf A, Flanagan A, McHenry BD, Altiok H, Smith PA, Harris GF, Krzak JJ. 2019 Segmental foot and ankle kinematic differences between rectus, planus, and cavus foot types. J. Biomech. **94**, 180-186. (10.1016/j.jbiomech.2019.07.032) 31420153

[57] McPoil TG et al. 2016 The use of a static measure to predict foot posture at midstance during walking. Foot **28**, 47-53. (10.1016/j.foot.2016.09.001) 27736722

[58] Baxter JR, Novack TA, Van Werkhoven H, Pennell DR, Piazza SJ. 2012 Ankle joint mechanics and foot proportions differ between human sprinters and non-sprinters. Proc. R. Soc. B **279**, 2018-2024. (10.1098/rspb.2011.2358) PMC3311895 22189400

[59] Raichlen DA, Armstrong H, Lieberman DE. 2011 Calcaneus length determines running economy: implications for endurance running performance in modern humans and Neandertals. J. Hum. Evol. **60**, 299-308. (10.1016/j.jhevol.2010.11.002) 21269660

[60] Scholz MN, Bobbert MF, van Soest AJ, Clark JR, van Heerden J. 2008 Running biomechanics: shorter heels, better economy. J. Exp. Biol. **211**, 3266-3271. (10.1242/jeb.018812) 18840660

[61] Honert EC, Bastas G, Zelik KE. 2020 Effects of toe length, foot arch length and toe joint axis on walking biomechanics. Hum. Mov. Sci. **70**, 102594. (10.1016/j.humov.2020.102594) 32217212

[62] Charles JP, Grant B, D'Août K, Bates KT. 2021 Foot anatomy, walking energetics, and the evolution of human bipedalism. J. Hum. Evol. **156**, 103014. (10.1016/j.jhevol.2021.103014) 34023575

[63] Honert EC, Bastas G, Zelik KE. 2018 Effect of toe joint stiffness and toe shape on walking biomechanics. Bioinspir. Biomim. **13**, 066007. (10.1088/1748-3190/aadf46) 30187893 PMC8777388

[64] Tanaka T, Suga T, Otsuka M, Misaki J, Miyake Y, Kudo S, Nagano A, Isaka T. 2017 Relationship between the length of the forefoot bones and performance in male sprinters. Scand. J. Med. Sci. Sports **27**, 1673-1680. (10.1111/sms.12857) 28207966

[65] Ueno H, Suga T, Takao K, Tanaka T, Misaki J, Miyake Y, Nagano A, Isaka T. 2018 Association between forefoot bone length and performance in male endurance runners. Int. J. Sports Med. **39**, 275-281. (10.1055/s-0043-123646) 29475206

[66] Nester CJ, Jarvis HL, Jones RK, Bowden PD, Liu A. 2014 Movement of the human foot in 100 pain free individuals aged 18–45: implications for understanding normal foot function. J. Foot Ankle Res. **7**, 51. (10.1186/s13047-014-0051-8) 25493100 PMC4260241

[67] Owings TM, Grabiner MD. 2003 Measuring step kinematic variability on an instrumented treadmill: how many steps are enough? J. Biomech. **36**, 1215-1218. (10.1016/S0021-9290(03)00108-8) 12831749

[68] McClymont J, Savage R, Pataky TC, Crompton RH, Charles J, Bates KT. 2021 Intra-subject sample size effects in plantar pressure analyses. PeerJ **9**, e11660. (10.7717/peerj.11660) 34221737 PMC8236230

[69] Fitzpatrick CK, Baldwin MA, Laz PJ, FitzPatrick DP, Lerner AL, Rullkoetter PJ. 2011 Development of a statistical shape model of the patellofemoral joint for investigating relationships between shape and function. J. Biomech. **44**, 2446-2452. (10.1016/j.jbiomech.2011.06.025) 21803359

[70] Nielsen RG, Rathleff MS, Moelgaard CM, Simonsen O, Kaalund S, Olesen CG, Christensen FB, Kersting UG. 2010 Video based analysis of dynamic midfoot function and its relationship with Foot Posture Index scores. Gait Posture **31**, 126-130. (10.1016/j.gaitpost.2009.09.012) 19854653

[71] Cavanagh PR, Morag E, Boulton AJM, Young MJ, Deffner KT, Pammer SE. 1997 The relationship of static foot structure to dynamic foot function. J. Biomech. **30**, 243-250. (10.1016/S0021-9290(96)00136-4) 9119823

[72] Lauder GV. 1995 On the inference of function from structure. In Functional morphology in vertebrate paleontology (ed. JJ Thomason), pp. 1-18. Cambridge, UK: Cambridge University Press.

[73] Kessler SE, Lichtwark GA, Welte LKM, Rainbow MJ, Kelly LA. 2020 Regulation of foot and ankle quasi-stiffness during human hopping across a range of frequencies. J. Biomech. **108**, 109853. (10.1016/j.jbiomech.2020.109853) 32636016

[74] Stearne SM, McDonald KA, Alderson JA, North I, Oxnard CE, Rubenson J. 2016 The foot's arch and the energetics of human locomotion. Scient. Rep. **6**, 19403. (10.1038/srep19403) PMC4726102 26783259

[75] Brown GL, Seethapathi N, Srinivasan M. 2021 A unified energy-optimality criterion predicts human navigation paths and speeds. Proc. Natl Acad. Sci. USA **118**, e2020327118. (10.1073/pnas.2020327118) 34266945 PMC8307777

[76] Hébert-Losier K, Finlayson SJ, Driller MW, Dubois B, Esculier J-F, Beaven CM. 2022 Metabolic and performance responses of male runners wearing 3 types of footwear: Nike Vaporfly 4%, Saucony Endorphin racing flats, and their own shoes. J. Sport Health Sci. **11**, 275-284. (10.1016/j.jshs.2020.11.012) 33264686 PMC9189709

[77] Grant TM, Diamond LE, Pizzolato C, Killen BA, Devaprakash D, Kelly L, Maharaj JN, Saxby DJ. 2020 Development and validation of statistical shape models of the primary functional bone segments of the foot. PeerJ **8**, e8397. (10.7717/peerj.8397) 32117607 PMC7006516

[78] Schuster R. 2023 Foot shape–function model data. Dryad Digital Repository. (10.5061/dryad.g4f4qrfwt)

[79] Schuster RW, Cresswell AG, Kelly LA. 2024 Human foot form and function: variable and versatile, yet sufficiently related to predict function from form. Figshare. (10.6084/m9.figshare.c.6978850) PMC10777145 38196364

